# Rodent Models of Developmental Ischemic Stroke for Translational Research: Strengths and Weaknesses

**DOI:** 10.1155/2019/5089321

**Published:** 2019-04-04

**Authors:** Mariangela Gennaro, Alessandro Mattiello, Tommaso Pizzorusso

**Affiliations:** ^1^Institute of Neuroscience, National Research Council (CNR), Via Moruzzi 1, I-56124 Pisa, Italy; ^2^Department of Clinical and Experimental Medicine, University of Pisa, via Savi 10, I-56126 Pisa, Italy; ^3^Department of Neuroscience, Psychology, Drug Research and Child Health NEUROFARBA, University of Florence, Area San Salvi–Pad. 26, I-50135 Florence, Italy

## Abstract

Cerebral ischemia can occur at any stage in life, but clinical consequences greatly differ depending on the developmental stage of the affected brain structures. Timing of the lesion occurrence seems to be critical, as it strongly interferes with neuronal circuit development and determines the way spontaneous plasticity takes place. Translational stroke research requires the use of animal models as they represent a reliable tool to understand the pathogenic mechanisms underlying the generation, progression, and pathological consequences of a stroke. Moreover, in vivo experiments are instrumental to investigate new therapeutic strategies and the best temporal window of intervention. Differently from adults, very few models of the human developmental stroke have been characterized, and most of them have been established in rodents. The models currently used provide a better understanding of the molecular factors involved in the effects of ischemia; however, they still hold many limitations due to matching developmental stages across different species and the complexity of the human disorder that hardly can be described by segregated variables. In this review, we summarize the key factors contributing to neonatal brain vulnerability to ischemic strokes and we provide an overview of the advantages and limitations of the currently available models to recapitulate different aspects of the human developmental stroke.

## 1. Introduction

An ischemic stroke is a transient or permanent interruption of the blood supply into the cerebral vasculature and represents worldwide one of the most important causes of death and of long-term disability in the survivors [1]. Although the risk of brain ischemia increases in the elderly, the insult can hit young people, at the perinatal and pediatric ages [2]. Depending on the developmental stage of the affected brain structures, a broad spectrum of clinical signs may arise [2] such as hemiplegic cerebral palsy that represents the most frequent deficit after developmental ischemia, with a prevalence of 90% within the affected children [1].

Despite several studies shedding light on different pathogenic mechanisms underlying the generation, progression, and pathological consequences of the developmental ischemic stroke, the translation from the bench to the bedside of these findings encounters several obstacles.

In translational research, animal models for strokes represent a fundamental tool (a) to understand the molecular mechanisms underlying the short- and long-term physiological responses of all individual neuronal systems and of the whole brain to injury, (b) to set up new therapeutic strategies to salvage and rescue those structures, and (c) to find the best temporal window of intervention with pharmacological and rehabilitation interventions [3, 4]. In this view, notwithstanding the complexity of all cascade events, the choice of a reliable model is a researcher priority to reconcile the existing marked differences between rodents and humans at the level both of the cerebral vasculature [5] and of the nervous system architecture [6]. Keeping in consideration of how hard it is to match developmental stages across different species, in this review, we aim to summarize developmental ischemic stroke pathophysiological mechanisms, focusing on key factors contributing to neonatal brain vulnerability. We also provide an overview of the models currently used to recapitulate the human developmental ischemic stroke, describing their advantages and limitations.

## 2. Clinical Features of Perinatal and Pediatric Ischemic Stroke

According to the timing of the stroke occurrence during development, two types of strokes are defined: perinatal and pediatric [2, 7]. The perinatal stroke, also known as neonatal, occurs from the 20th week of fetal life through to the 28th postnatal day and represents a significant cause of death and disability involving as many as 1 in 2,300 live births [1, 7]. By contrast, with a prevalence of 2-13 in 100,000, the pediatric stroke can occur from the twenty-eighth day after birth through to age eighteen [8–12]. Despite their different etiology, ischemia due to vascular (arterial or venous) thrombosis is the main cause of hemiplegia in up to 94% of cases of the perinatal versus pediatric stroke [1, 2, 13–15]. Additional neurological signs including intellectual disabilities, behavioral deficits, language and visual defects, psychiatric disorders, and epilepsy are more frequent after the perinatal stroke with respect to the pediatric condition [1, 2, 7, 9, 14, 16–18].

As stated before, depending on the timing of ischemia occurrence, different structures can undergo prevalent damage. For example, in preterm injured babies, white matter injury is more affected due to the abundance of developing oligodendrocytes that are highly sensitive to excitotoxicity and neuroinflammation [19]. On the other hand, in term babies, who have significantly less oligodendrocyte progenitors, grey matter structures (e.g., the basal ganglia, thalamic nuclei, and cerebral cortex) are the most commonly affected by the injury [20, 21]. In general, the perinatal stroke seems to be associated with a greater risk of worse outcomes [2, 7, 14, 18] when compared to the pediatric stroke scenario. This phenomenon is linked to the existence of different stages of the critical period throughout development in which the brain is differently susceptible to the early damage [2, 7]. Thus, in contrast to “Kennard's principle” by which the younger brain holds a greater capability to recover from injury, it seems that an earlier injury may in some cases more deeply impact the early developing brain, finally disturbing and so disrupting its pattern of maturation. This form of plasticity called maladaptive plasticity could be particularly disruptive for motor circuitry refinement where an aberrant mechanism of plasticity frequently arises [2, 7, 22–25]. Under maladaptation, the affected corticospinal tract does not exert the usual role in the movement control proper of the first few months after birth [24], but rather, an abnormal bilateral pattern of the innervation of the spinal motor neuron is observed, with deleterious consequences for long-term motor function [22–24, 26]. Perinatal and pediatric strokes have long remained undiagnosed or misdiagnosed, because of the difficulty of interpreting the paucity of motor handicaps [7]. In this context, Eyre and others in 2007 suggested that the delay in the emergence of motor signs depended upon the activity-dependent competition between the ipsilateral corticospinal tract (CST) from the undamaged side and the spared CST axons from the damaged side. However, recent efforts in clinical research have been made to find novel tools to detect hemiplegic signs as early as possible. For instance, the assessment of general movements at the neonatal epoch has been pointed out as a promising predictive method to detect the presence of neonatal cerebral infarction in infants [27, 28].

## 3. Ischemic Stroke Pathophysiology in the Immature Brain

**Figure 1:**
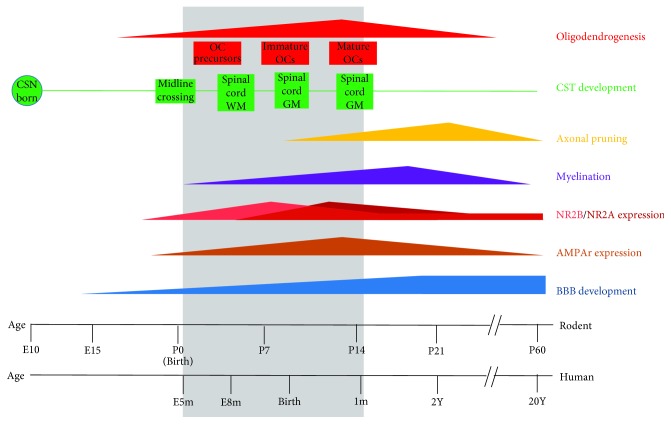
Comparison between the rodent and human development of some molecular, cellular, and structural elements of the nervous system. The grey rectangle depicts the “perinatal” range throughout life. Perinatal insults, such as an ischemic stroke, that hit during this age can interfere with several aspects of neural development.

Several experimental and clinical studies have been reviewed on the pathophysiology of perinatal and pediatric ischemic strokes frequently showing the presence of different mechanisms activated upon developmental injury [2, 14, 29]. The severity of damage following the neonatal brain ischemia may depend upon several factors: the type of neuronal cell death mainly activated during development [30], the maturation of the immune system [31], and the developmental stage of the cerebral vasculature [32] (Figure 1).

### 3.1. Excitotoxicity

Soon after blood flow interruption in the territory of a major brain artery, a failure in energy-dependent processes is generated, with the sudden loss of membrane potential, strong depolarization and Ca^++^ influx due to the activation [33]. As a consequence, neurons and glia undergo ion and water imbalance with the subsequent formation of intracellular edemas and membrane depolarization that leads to glutamate-dependent excitotoxicity that in turn triggers alteration in the brain metabolic profile [34] and death pathways [35]. The immature brain shows unique patterns of cell death activation in response to an ischemic lesion [36–38]. In fact, while necrosis is the prominent mechanism of neuronal cell death in the core lesion in adults, apoptosis is more readily activated in the immature brain. This is in part due to the high expression of key components of apoptotic pathways, such as caspase-3, that have a pivotal role in the programmed neuronal death during brain development [30, 36, 37]. Indeed, in a developing rat model of hypoxia-ischemia (HI), AMP-activated protein kinase (AMPK), a sensor of cellular energy status also involved in chronic inflammatory disorder [39], regulates FOXO3a-mediated neuronal apoptosis through increased expression levels of pro-apoptosis proteins, such as Bim and Caspase-3 [40].

The immature brain displays high excitability that can contribute to excitotoxic injury. This intrinsic high excitability of the immature brain relies mostly on a developmental increase in expression levels of the glutamate receptor [41, 42], both in ionotropic (NMDA) and in metabotropic (AMPA) ones. In fact, experimental evidence in rats suggests that the NMDA receptor density peaks late in the first postnatal week in both the hippocampus and the neocortex, whereas the AMPA receptor density peaks in the second postnatal week at around P10 [43]. Moreover, a different composition of individual receptor subunits of NMDA [42, 44] and AMPA [45] due to a developmental regulation of their expression also contributes to increasing the glutamate-dependent excitotoxicity after a perinatal ischemic lesion. The higher expression of NR2B versus NR2A, together with a lower ratio of the GluR2 expression versus other AMPA receptor subunits in the immature neocortex and hippocampus, accounts for increased Ca2+ permeability, which in turn leads to exacerbated excitotoxicity after the injury [46]. An additional factor impinging on glutamate-dependent excitotoxicity after early injury is the intrinsic nature in action of the GABAergic system, which is immature and excitatory during early postnatal brain development [47, 48]. The reduced expression of several endogenous antioxidant enzymes as well as the very high concentration of unsaturated fatty acids, the high rate of oxygen consumption, and the availability of redox-active iron [49] also contributes to cytotoxicity.

### 3.2. Inflammation

Free-radical formation and activation of the inflammatory cascades also contribute to neuronal cell death after an ischemic injury in the immature brain [29]. Inflammation plays a dual role in perinatal ischemic stroke pathophysiology [50]. It represents a risk factor of perinatal stroke onset; however, it also contributes to protect the brain from injury, by supporting tissue healing [51, 52]. Its detrimental effects could be due to the facilitatory effects of perinatal inflammation on the pathophysiology of ischemia [53, 54], an effect linked to the ability of congenital inflammation eliciting thrombus formation; for a review, see [55]. The immaturity of the immune system at the perinatal age also impinges on the brain pathological response to ischemia [50]. For example, the nonclassical complement activation in term infants as well as in rat pups is downregulated with respect to the mature brain [56]. Furthermore, in adulthood, microglia activation plays a detrimental role in the acute phase of the ischemic lesion as it produces inflammatory mediators such as ROS and releases other toxic molecules [54]. In contrast, during development, microglia can play a reparative role [57, 58], since it actively releases anti-inflammatory cytokines and neurotrophic factors that contribute to resolve inflammation processes protecting viable neurons from apoptotic death [59]. Direct evidence of its protective role comes from two experimental studies where selective pharmacologic depletion of microglial cells two days before inducing tMCAO in P7 rats caused, respectively, a larger infarct size [59] and increased intracerebral hemorrhages [60]. Astrocytes act in concert with microglia in neonatal stroke pathophysiology. Indeed, early after injury, astrocytes actively contribute to the production of proinflammatory cytokines, in association with neurons and endothelial cells [59]. As for an adult stroke, also after neonatal ischemia, astrogliosis is sustained by higher activation of JAK/STAT signaling in both astrocytes and neurons, with a final insulting effect on brain cells [61–63]. In this context, recent work demonstrated that reducing this signaling pathway indirectly either by inhibiting the STAT3 transducer and activator glycogen synthase kinase 3*β* (GSK3*β*) [61] or by blocking JAK2 and downstream STAT3 phosphorylation [63] promotes neuroprotection and reduced inflammatory response after a neonatal stroke. However, other controversial result come from a study carried in a model of hypoxia-ischemia (HI), where it has been shown that reactive astroglia does not exacerbate lesion extension, since GFAP deletion did not affect infarct volume [64]. Similar results were observed in a model of perinatal brain injury [65]. In the chronic phase, astrocytes contribute to limit edema after neonatal brain injury, since astrocyte end-feet in the neurovascular unit increases aquaporin 4 expression, thus facilitating water clearance to the vascular compartment [65].

### 3.3. Immaturity

Another crucial intrinsic factor contributing to the higher vulnerability of the developing brain to neonatal ischemia is the immaturity of brain microvessels [66]. For example, comparison of protein and transcript contents of the mouse forebrain enriched in microvessels at different ages across development showed an age-dependent increase of proteins and mRNA specific for endothelial cell adhesion, junction pathway, and extracellular matrix as well as for a shift of energy metabolism, transport, and antioxidant effector proteins, all associated with the acquisition of a mature microvessel structure [66]. Brain-blood barrier (BBB) permeability also appears different when compared with the adults both in physiological and pathological conditions [67]. In fact, BBB permeability in the early postnatal age is much lower with respect to the later stage of development, and in response to perinatal ischemic injury, extravasation of albumin at 2 hours after reperfusion is increased from 5- to 25-fold in the rat adult injured brain but only 2-fold in a newborn [67]. It has been proposed that the reduced BBB permeability at the early stage of brain development relies upon a higher expression of several tight junction and basal membrane components in neonates [67], on distinct mechanisms of endothelial cell activation, immature extracellular matrix (ECM) components [66], and neutrophil-endothelial interactions [67, 68]. Altogether, these mechanisms, in addition to preserving BBB integrity, also prevent neutrophil, monocyte, and T and B cell infiltration from the peripheral district to the brain [32]. Taking together, all these data point at the existence of a critical time window of neonate brain vulnerability to early damage that strongly determines the pattern of brain injury.

## 4. Developmental Ischemic Stroke Models

While several animal models of the adult ischemic stroke have been developed so far, few animal models of perinatal and pediatric strokes are available to recapitulate the mechanisms underlying the onset and the evolution of acute and long lasting deficits in children. In Table 1 a summary of the rodent models of the developmental ischemic stroke, and their assessment, is listed.

### 4.1. Models of Hypoxia-Ischemia

Over the past three decades, the Levine-Rice model of neonatal hypoxic-ischemic (HI) has been extensively used to generate the human perinatal ischemic stroke and has been characterized through histological analysis as well as behavioral tests (for reviews, see [69–71]). This model is a modification in the pups of the Levine preparation previously performed in the adult rat [72], and it is characterized by one to more hours of unilateral ligation of the common carotid artery followed by reperfusion and recovery. Afterward, whole body hypoxia is practiced by placing animals into a hypoxic chamber containing humidified 8% O_2_. This model causes hypoperfusion in the ligated side of the brain, while the nonligated side is exposed to hypoxia alone [73]. Rat pups at P7 have been preferentially used versus mice [74, 75] to study neonatal stroke pathophysiology [14, 55], as well as neuroprotection, regenerative potential of the immature brain [76–79], and the applicable rehabilitative therapies [80]. However, the HI neonatal model generates high variability in infarct size, leading to a multifactorial pathological condition; moreover, model induction strikingly differs from the etiology of hypoxic-ischemic injury in humans and does not cause a consistent focal injury pattern, making study of the injured core and penumbra more challenging [74, 75].

### 4.2. Models of Occlusion of the Middle Cerebral Artery

Since human perinatal ischemic strokes mainly affect the MCA [81, 82], models developed for adult ischemic stroke were adapted to earlier ages. The MCAO model implies the temporary occlusion of the common carotid artery (CCA), introducing a suture directly into the internal carotid artery (ICA) and advancing the suture until it interrupts blood flow to the MCA [83, 84]. Depending on the duration of MCAO, interruption of cerebral blood flow CBF can be transient or permanent, causing therefore mild to severe brain damage and outcome [83]. Furthermore, not only the infarct size but also reperfusion can be modulated depending on the duration of occlusion [74]. Temporary MCAO in neonatal animals was investigated for the first time by Ashwal et al. [85], who performed this technique in P14-P18 rats. 3 hours of occlusion induced a lesion that affects 40-50% of the total hemisphere, resembling in part a global human pediatric stroke. MCAO was also performed in P7 rats, where disruption of CBF and cytotoxic edema formation were observed in MCA territory, accompanied by subsequent microglia and astroglia infiltration after reperfusion [86]. Unfortunately, this method produced a high mortality rate, with only 21% of rats still surviving after 28 days [87], making difficult any long-term assessment of outcomes. Embolic MCAO was also implemented [86]: the embolus measure was designed according to the rat size and resulted in an infarct affecting 51-56% of the ipsilateral hemisphere [88]. Ninety minutes of the intraluminal filament MCAO model at P20-25, followed by 22 h of reperfusion, was also used to characterize a mouse model of a childhood ischemic stroke [89]. One of the most interesting data obtained in this study is the assessment of sex-specific responses to cerebral ischemia in a juvenile mouse brain. The results showed a lack of gender difference in the response to ischemic injury and a sexual dimorphic neuroprotective role of estrogen [89]. These results greatly differ from what is usually observed in adults, either in humans or in rodent models [90].

Transient MCAO without permanent ligation or cauterization has been applied to P10 rats, comparing the effect of different durations of artery occlusion on the extension of brain injury and on behavioral outcome. In this case, extension of the brain lesion correlated with duration of occlusion, since a 90 min occlusion produced a mild-to-moderate injury pattern affecting the striatum and causing transient locomotor deficits, while 3 h caused a moderate-to-severe injury that often affected the cortex and hippocampus and caused enduring locomotor deficits that outlasted the reperfusion phase [91]. Recently, direct electrocoagulation of the unilateral MCA in P12 CB-17 mice has been used: this model holds a reduced variability both in brain injury and in CBF after 24 h from insult with respect to the HI model. Furthermore, using electrocoagulation as a permanent insult, significant neurofunctional deficits in the rotarod and open field can be elicited [92].

### 4.3. Models of Thrombotic Ischemia

The photothrombotic stroke is a model of thromboischemia based on intravascular photooxidation of a photoactive dye (in most cases, the rose bengal given through intraperitoneal administration) through brief irradiation of the intact skull by a light beam at a specific wavelength [93]. Depending on the intensity and duration of light illumination, as well as the stereotaxic coordinates chosen, different extensions of the lesion can be produced [6, 94]. Until now, photothrombotic models have been mostly used to study stroke in adults [95–98], and only recently, it has been used to recapitulate the perinatal stroke condition both in neonate piglets [99] and in rats at P7 [100]. Among the advantages of this model is the possibility of creating small size infarcts to target specific regions [6]. However, there are intrinsic disadvantages of this model since, in contrast with human stroke pathophysiology, its nature is only occlusive, and no growth and maturation of the ischemic penumbra and local collateral flow/reperfusion can take place [101].

### 4.4. Models of ET1 Vasoconstriction

Endothelin 1 (ET1) is a small (21 amino acids) vasoactive peptide produced by the endothelium and smooth muscle cells [102] which acts as a paracrine and autocrine factor [103] constricting vessels [104] through specific receptors (ETRA and ETRB) [105]. ETRA is mainly located on smooth muscle cells, and its activation is thought to be the major contributor to vasoconstriction upon ET1 binding [106]. Instead, ETRB is localized on both the smooth muscle and endothelial cells but is associated with vasodilation, caused by the release of nitric oxide (NO) and prostacyclin from endothelial cells [107]. Other than in vascular cells, the endothelin system (ET system) is also present in the central nervous system [102], where it plays an important role in the case of lesion occurrence. Indeed, after brain injury, ET1 is acutely overexpressed in the cerebrospinal fluid and plasma of humans [108, 109], rats [110], and pigs [111], suggesting that endogenous upregulation is an evolutionary conserved mechanism. However, whether the ET system overactivation may be protective or detrimental for the postlesion outcome is still a matter of debate. Several experimental works indicate that the endogenous ET system upregulation may contribute to lesion pathophysiology. Indeed, postlesion upregulation of either ET1 or ETR expressions correlate with astrogliosis [112], extent of the brain lesion [113], BBB dysfunction [114–116], and inflammation [117]. This evidence is a very important issue to keep in mind when generating ET1 models of ischemia, as it influences the interpretation of experimental results. ET1 can be either stereotaxically injected into parenchymal regions of interest or topically applied on the pial surface of the brain, to constrict local arterioles, or near the MCA [118, 119] reperfusion occurs, but at a much slower rate with respect to the intraluminal suture model. Lesion size can be modulated by varying the concentration or volume of ET1 to achieve reproducible injury [120]. The constant hypoperfusion rate prevents the development of an extensive edema, moving partially away from the human ischemia. On the other hand, this model seems to be more appropriate for chronic long-term studies rather than for studies on the acute effects of a stroke [121].

In contrast to adult stroke studies, very few works have used ET1 to generate models of the developmental stroke thus far [122]. ET1 was previously injected into the striatal area of the juvenile (P21) rat brain to induce a reproducible focal lesion [123], but only anatomical changes in response to ET1 injection were evaluated. Tsenov et al. in 2007 [124] used intrahippocampal ET-1 injection to generate a model of ischemia-induced seizures in immature rats, at P12 and P25, respectively, showing that at both developmental ages, ET1 into the dorsal hippocampus elicited convulsions as well as neuron loss.

### 4.5. Rodent Models: Similarities and Differences with Human Brain Development

The success of generating reliable models of the human developmental stroke strongly relies upon the ability to get the similarities in the cross-species corticospinal system function and development (for a review, see [125]). Most of the studies use rodent models because they can be easily manipulated to explore the genetic basis of motor development [126] as well as to understand motor learning mechanisms using behavioral and functional approaches [127]. Rodents show some similarities with humans at the CST level [127–129].

Indeed, as in humans, rodents have a CST that projects the full length of the spinal cord [129–131] and is involved in fine movement control [127, 132]. Both in humans and in rodents, CST development is accomplished at the postnatal age [133, 134]. Indeed, temporal changes in the diffusion anisotropy quantified by diffusor tensor imaging DTI in rats from P0 (day of birth) to P56 showed developmental changes in the DTI metrics in multiple gray and white matter structures related to neuronal and axonal pruning and myelination [133]. Furthermore, in the neonatal rat, the corticospinal projection originates from the whole neocortex including the visual cortex, and corticospinal projections also have transient ipsilateral projections that are predominantly pruned when maturity is reached [135].

However, notable differences between the human and rodent developing brain exist. *In primis*, there is a complete absence of gyrification in the rodent brain [136]; second, great differences in the CST path are present: rodent CST axons run into the dorsal funiculus and do not establish direct synapses onto spinal motor neurons [137], but rather, the CST almost entirely projects more dorsally to the premotor spinal circuits [134, 138–142]. Concerning brain vasculature, similarities and differences of the circle of Willis between rodents and humans have been reported [5]. Both in rodents and humans, the internal carotid artery irrorates the anterior circulation whilst the posterior cerebral artery supplies the posterior circulation [5]. Moreover, in both species, the internal carotid artery provides the major blood source to the encephalon; however, it is more extended in rodents versus humans since it has a greater number of collaterals which supply a wide cerebral area [5]. This interspecies difference in brain vascular morphology may impinge on the degree of blood flow deprivation induced by different models and accordingly on the entity of neuronal damage.

### 4.6. Milestones Controlling CST Development across Different Species

**Figure 2:**
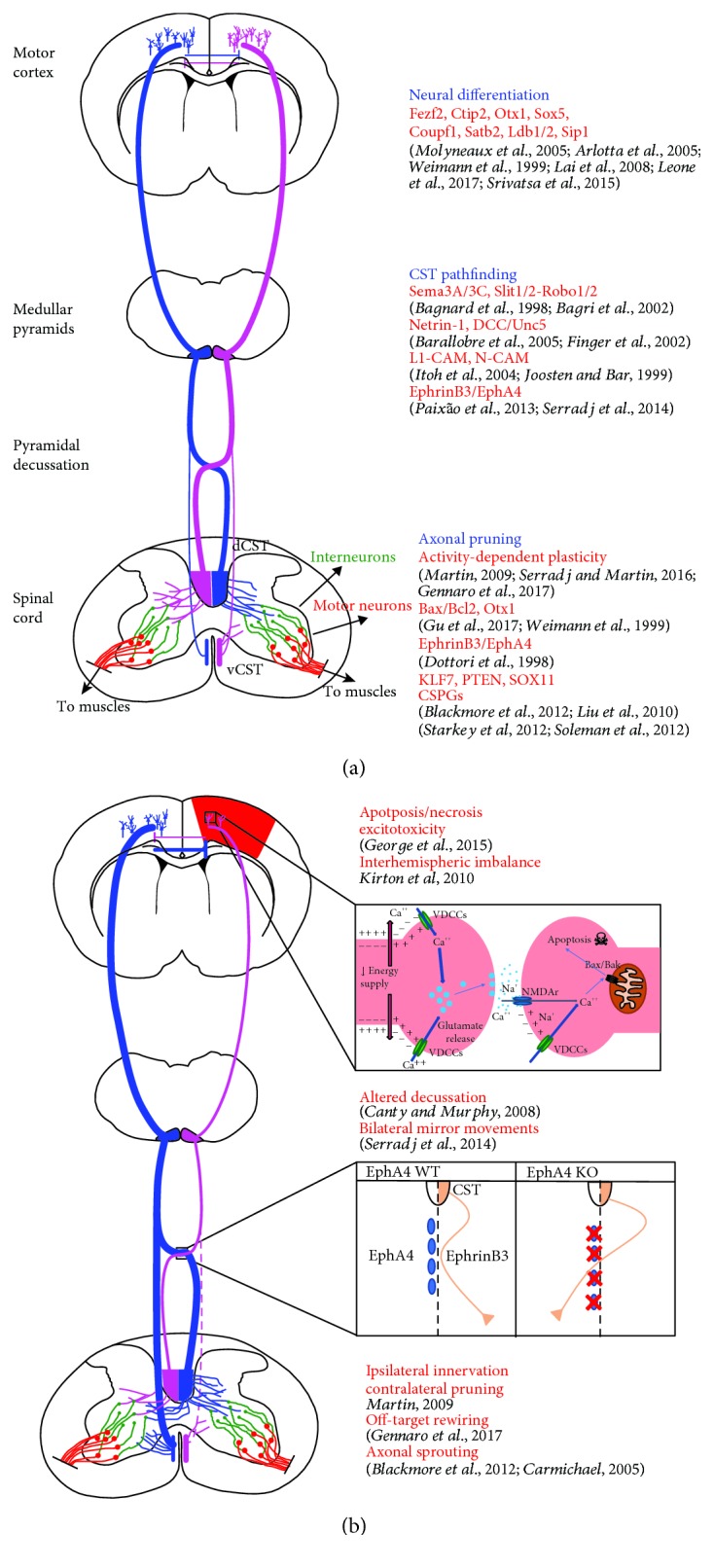
(a) Molecular and environmental factors involved in physiological CST development in rodents. (b) Processes altered after a brain injury that hits during CST development. Insets show some of the mechanisms involved in the acute damage provoked by cerebral ischemia (excitotoxicity, top right) and the factor involved in the axonal pathfinding and midline crossing in the CST development (EphA4/EphrinB3, bottom right).

Another crucial factor to be kept in mind when generating a rodent model of experimental models of a stroke is the ability to match the age-specific motor behavior repertoire with the progressive steps of CST maturation across species. While the corticospinal system matures, adaptive motor behaviors begin to be expressed [143, 144]. In mammals, CST development begins prenatally while mature motor skills are developed during the first month in the rat [145] and the first 2 to 3 months in cats [25]. Human motor development is incomplete until 12-13 years [146, 147]. As shown in Figure 2, several experimental studies have clarified that the mammal CST maturation process involves the interplay between genetics, neural activity, and experience to allow appropriate circuit formation and acquisition of complex tasks [6, 122, 134, 148–176]. For example, guidance cues such as EphrinB3 and its receptor tyrosine kinase EphA4 ensure the correct CST pathfinding [172], since selective elimination of the EphA4 gene in the mouse forebrain leads to a strong CST bilateral projection to the spinal cord that persists up to adulthood with enduring skilled motor impairments (Figure 2) [168]. Activity- and use-dependent processes subsequently shape the pattern initially established by genetic mechanisms and lead to the withdrawal of less active ipsilateral CST projections while contralateral ones are instead reinforced [23, 24, 141, 166]. Indeed, studies in cats have revealed that blocking motor experience or motor cortex activity causes defects in CS axon remodeling in the spinal cord, leading to permanent impairments in skilled movements [177]. Furthermore, concurrently to CS axon remodeling, motor maps for interjoint muscle synergies also develop during the postnatal stages in cats [155]. Recently, the mechanism by which rodents gradually acquire precise control over their flexor and extensor muscles to allow acquisition of skilled abilities has been elucidated [178]. In this elegant work, Gu et al. showed that maturation of muscle activation patterns controlling skilled movements is acquired through reorganization of the CS axons controlling antagonist muscles, according to an activity-dependent Bax/Bak-caspase pathway. Deletion of the Bax/Bak proteins selectively in the mouse motor cortex resulted in the lack of activity-dependent pruning of exuberant axon collaterals [178], suggesting therefore the nonapoptotic pathway Bax/Bak as a novel milestone for proper CST motor development in rodents. Thus, across species, motor control development implies a triad of events during the refinement period: loss of transient ipsilateral termination with growth of experience-selected axons to local spinal targets, development of motor cortical motor maps, and finally myelination [179].

Although great insights into the milestones controlling normal maturation of CST across different species have been achieved, a debate on the appropriate matching of age between human and rodent neonates, as well as on how to correlate neuronal events that occur during maturation across these species, still remains open [180]. Some authors suggest that depending upon different criteria, such as brain weight growth [181], white matter myelination [182], corticospinal system development [183], and EEG maturation [184], the human term would include P7-P10 in rodents, with brain development at P7 in rats being more comparable to that of premature or full-term infants [70, 182, 185]. P20 in rodents would correspond to a 2–3-year-old human child [180, 181]. Nonetheless, there are some controversial opinions about which postnatal age in the rodent would recapitulate the term infant stage. For example, in an attempt to generate a model of the human term moderate HI, Quairiaux et al. used rat pups at P3 to characterize the effect of this really early damage on morphological and functional outcome [186]. In this work, in agreement with previous findings [187], the lesion at this early developmental stage caused impairments that mainly involved the somatosensory parietal cortex [186]. The importance of age of ischemia occurrence as a determinant for stroke outcome is underscored by a study that compared the effects of a stroke in the rat motor cortex at two temporally close ages: P14, when CS axons reach a maximum level of spinal cord gray matter innervations [154], and P21, when the CST axon pruning reaches its maximal levels [188]. Focal ischemic lesions at these two ages caused substantially different outcomes: the P14 lesion resulted in being more detrimental than the P21 lesion for long-term motor outcome in association with an extensive but mistargeted CST sprouting at the spinal cord level [122]. These data imply the existence of a strict age-dependent regulation of CST plasticity that can even be maladaptive during development.

## 5. Conclusions

Despite the variability in the techniques adopted and the developmental stages used to model human developmental ischemic strokes, preclinical studies continue to be extremely useful. Indeed, they inform us about the existence of multiple factors influencing the postinjury functional outcome. The timing of lesion occurrence seems to be critical, as it strongly interferes with CST development and determines the way spontaneous plasticity takes place. Classical studies showed that the effects of visual deprivation during temporal windows of development-designated critical periods dramatically impaired visual acuity maturation resulting in amblyopia. Similarly, a developmental brain injury causing a “deprivation” of activity of CST could also have long-term functional consequences that could strongly depend upon the age of the lesion and the relationship with critical motor periods [23]. The comparison with the current knowledge coming from visual system experience-dependent development suggests that experience-dependent changes could also be exploited to open a window for restorative therapies in the case of early motor system injuries. So far, harnessing poststroke neural plasticity via electrophysiological and behavioral approaches was found to have beneficial effects promoting significant recovery of motor function, and early intervention after a perinatal ischemic stroke has been shown crucial in preventing maladaptive plasticity [22, 122]. However, future studies should be directed to identify the age-specific molecular programs triggered by developmental injury. Specifically, finding a causal link of the age-specific regulation between genetic factors and environmental molecular cues would help to determine the pattern of sprouting and therefore implement more effective therapeutic strategies aimed at regaining or preserving motor functions. Technological development has dramatically accelerated moving towards cell-specific studies, both at the molecular (e.g., single-cell sequencing from defined populations) and functional (e.g., in-depth in vivo functional imaging and noninvasive stimulation) level. Applying these methods to selectively study the CST and its milieu in models of a juvenile stroke will be fundamental to understand which molecular factors and which pattern of electrical activity can regulate developing CST growth and pruning, with positive consequences on the development of treatments that could also be beneficial in adult models of CST lesions.

## Figures and Tables

**Table 1:** Summary of main rodent models of the developmental stroke used in translational research.

Animal	Age of lesion induction	Method of induction	Age of assessment	Variables assessed
Rat [40, 49, 63, 73, 79, 80, 180–183, 185, 186]; mouse [49, 61, 63, 64]	P7 [40, 49, 63, 73, 80, 180–183, 185, 186]; P9 [61, 64]; P10 [79]; P3 [175]	Hypoxia-ischemia based on the Levine-Rice method	Up to P11 [189]; P9 [40, 73, 181, 183]; P8 [190]; up to P67 [191]; P12 [63, 175, 182]; P21 [175, 185]; within 3 hr after lesion [186]; P11 and P40 [79]; from P21 to P60 [80]; up to P10 [40]; 6 hr post-HI and at P16 [61]; P31 [64]	Analysis of damage by MRI [73, 189]; analysis of brain edema by histology [192]; behavioral assessment of sensorimotor function [73]; analysis of intracellular calcium accumulation [190]; phosphocreatine, neuronal MAP-2, SNAP-25, and glial GFAP [193]; analysis of lesion volume and of white matter injury by histology [49, 61, 73, 187, 191, 192, 194]; analysis of systemic physiological variables (mean arterial blood pressure, heart rate, PO_2_, PCO_2_, pH, lactate, and glucose) and of high-energy phosphate and glycolytic intermediates [195]; effects of adiponectin treatment efficacy on the brain infarct area, apoptosis, brain atrophy, and neurological function [79]; investigation of efficacy of combining constraint-induced movement therapy (CIMT) and electroacupuncture on motor asymmetry and on lesion size and astrogliosis [80]; analysis of the role of AMPK signaling in the developing rat brain with HI [40]; analysis of inflammatory activation by immunohistochemistry [187]; assessment of oxidative stress after injury [49]; assessment of JAK/STAT signaling in brain inflammation [61, 63] and neuroprotection [63] by biochemical, molecular, and histological approaches [61, 63]; role of GFAP deletion on astrogliosis after HI and on infarct volume by immunohistochemistry [64]

Rat	P7	Embolus MCAO	Up to P8	Analysis of lesion volume by histology [88]

Rat	P7	MCA electrocoagulation associated with 1-hour left CCAO	Up to P90	Analysis of inflammatory responses by histology [196]

Rat [59, 65, 85, 86, 91, 188, 189]; mouse [60]	P14-P18 [85]; P7 [30, 59, 60, 67, 86, 188, 189]; P10 [65, 91]	Transient MCAO	P8 [85]; up to P90 [188]; P8 [67] up to P10 [60, 189]; P8 [30]; P10 [91]; P8 and P10 [59]; up to P38 [65]	Analysis of lesion volume by histology [59, 65, 85, 86, 91, 197, 198]; analysis of lesion evolution by MRI [59, 65, 67] and neuroprotection assessment [30, 91]; microglia activation by histology [59]; BBB integrity postinjury by histological, biochemical, and molecular techniques [67]; assessment of the role of microglia on hemorrhages by histological, biochemical, and molecular techniques [60]; assessment of brain edema through brain aquaporin-4 expression profiling [65]

Mouse [30, 89, 90]	P12 [92]; P7 [30]; P20-25 [89]	MCAO	Up to P68 [90]; P8 [30]; 22 hr after lesion [89]	Analysis of lesion volume by histology and behavioral assessment of functional deficits [92]; anatomical analysis of caspase-3 activation in the ischemic core and penumbra [30]; effects of ischemia and estrogen treatment on the proapoptotic gene Bax [89]

Mouse [76, 77]	From P3 to P10 [76]; from P3 to P10 [77]	Chronic hypoxia	P10 and P48 [76]; P18 and P48 [77]	Analysis of injury by histology and unbiased stereological analysis of neurogenesis by BrdU assay [76, 77]

Rat	P7	Photothrombosis	P12 and P25	Study of PTZ-seizure susceptibility by EEG recordings [100]

Rat [103–105]	P14 [103]; P21 [103, 104]; P12 and P25 [105]	ET1 injection: intracortical [103]; intrastriatal [104]; intrahippocampal [105]	From P60 [103]; not specified [104]; up to 22 hr postinjury [105]	Assessment of lesion timing on damage volume, long-term motor outcome, and axonal sprouting of contralesional CST at the red nucleus and spinal cord level using anterograde tracing [122]; MRI analysis of damage extension, CBF volume and metabolic changes, and BBB integrity [123]; assessment of ischemia-induced seizures by video/EEG recordings [124]
